# Navigating ethics in HIV data and biomaterial management within Black, African, and Caribbean communities in Canada

**DOI:** 10.1186/s12910-025-01161-0

**Published:** 2025-01-16

**Authors:** Rusty Souleymanov, Bolaji Akinyele-Akanbi, Chinyere Njeze, Patricia Ukoli, Paula Migliardi, Linda Larcombe, Gayle Restall, Laurie Ringaert, Michael Payne, John Kim, Wangari Tharao, Ayn Wilcox

**Affiliations:** 1 Faculty of Social Work, University of Manitoba, 173 Dafoe Road West, Tier Building, office 500 C, Winnipeg, Winnipeg, MB R3T 2N2 Canada; 2 Department of Community Health Sciences, Rady Faculty of Health Sciences, University of Manitoba, Winnipeg, MB Canada; 3 Winnipeg Regional Health Authority, Winnipeg, MB Canada; 4 Department of Internal Medicine, Rady Faculty of Health Sciences, University of Manitoba, Winnipeg, MB Canada; 5 Department of Occupational Therapy, Rady Faculty of Health Sciences, University of Manitoba, Winnipeg, MB Canada; 6 Nine Circles Community Health Centre, Winnipeg, MB Canada; 7 Manitoba HIV-STBBI Collective Impact Network, Winnipeg, MB Canada; 8 National Laboratory for HIV Reference Services, Public Health Agency of Canada, Winnipeg, MB Canada; 9 Women’s Health in Women’s Hands Community Health Centre, Toronto, ON Canada; 10 Klinic Community Health, Winnipeg, MB Canada

**Keywords:** HIV community-based testing, Ethical concerns, Ethical considerations, Health data, Secondary use of biomaterials

## Abstract

**Background:**

This study explored the ethical issues associated with community-based HIV testing among African, Caribbean, and Black (ACB) populations in Canada, focusing on their perceptions of consent, privacy, and the management of HIV-related data and bio-samples.

**Methods:**

A qualitative community-based participatory research (CBPR) approach was employed to actively engage ACB community members in shaping the research process. The design included in-depth qualitative interviews with 33 ACB community members in Manitoba, Canada. The study was guided by a Community Guiding Circle, which contributed to study design, data analysis, and interpretation. A diverse sample was recruited through community agencies, social media, and flyers, with considerations for variations in age, gender, sexual orientation, and geographical location. The study employed iterative inductive thematic data analysis.

**Findings:**

Participants expressed significant concerns about the collection, sharing, and use of HIV data from healthcare encounters, revealing mistrust towards institutions like police, child welfare, and immigration accessing their health information. Their worries centered on the handling of biological samples, data misuse, potential human rights violations, HIV criminalization, deportations, challenging consent, privacy, and bodily autonomy principles. While open to contributing to medical research, they unanimously demanded greater transparency, informed consent, and control over the secondary use of their health data.

**Conclusions:**

The study underscores the need for culturally safe approaches in HIV testing and ethical governance in healthcare for ACB communities. It highlights the importance of prioritizing participant empowerment, ensuring transparency, practicing informed consent, and implementing robust data security measures to balance effective HIV information management with the protection of individual rights.

**Supplementary Information:**

The online version contains supplementary material available at 10.1186/s12910-025-01161-0.

## Introduction

This study explored the ethical issues in community-based HIV testing among African, Caribbean, and Black (ACB) communities in Canada, a key priority population in HIV research and policy [1–7]. Delving into the ethics of HIV testing within ACB communities requires a multifaceted exploration that considers historical legacies, community dynamics, and the fundamental tenets of justice, equity, and human rights [8]. Historical healthcare injustices, like the Tuskegee study, forced sterilizations, and unethical medical experiments, have bred mistrust in medical research and testing among ACB people [8, 9]. Thus, the narrative of the ethics of community-based HIV testing cannot be isolated from the context of historical exploitation and injustices faced by Black individuals within the realm of healthcare and the current experiences of anti-Black racism [9]. Paul Farmer’s concept of structural violence [10, 11] sheds light on the systemic and racial disparities in HIV testing within ACB communities, highlighting the importance of examining the interplay between ethical considerations and the socio-structural inequities encountered by these communities.

Ethical issues in HIV testing, as documented in the literature [12–14], have not been extensively explored within ACB communities. Key ethical challenges in HIV testing include conflicting mandatory reporting and confidentiality [15], ethical dilemmas for healthcare professionals balancing individual rights and public health [16], and issues around voluntary informed consent [14, 17]. Despite this existing research, significant gaps persist in comprehending the ethical complexities unique to community-based HIV testing within ACB communities. These complexities are also not thoroughly addressed by ethics review boards at community agencies.

In Canada, HIV test samples are processed by local or national labs, with results shared with healthcare providers and public health departments [18]. Opt-out HIV testing is generally not implemented; instead, most provinces require specific informed consent for HIV testing [19]. HIV testing and the management of resulting data are governed by complex policies that intersect with mandatory reporting, privacy regulations, and surveillance mechanisms [19–21]. Canadian laws mandate that positive HIV test results must be reported to public health authorities to ensure effective disease monitoring and control, though the extent to which identifiable data is shared varies by province and jurisdiction [3, 6, 18–21]. In certain cases, information can also be accessed by healthcare providers and public health departments [18–21]. Practices surrounding data sharing with non-health entities such as insurance companies, law enforcement, or immigration authorities remains less clear.

While crucial for public health, digital health system trends raise surveillance and privacy concerns [20], especially in Black communities [21]. Concerns include excessive surveillance, privacy breaches, and racial profiling [21]. Advanced surveillance technologies have been shown to disproportionately affect Black communities, increasing risks of unintended data use and discriminatory impacts [22]. Specifically, data retention for research, such as re-use of HIV surveillance data in retrospective analyses, poses distinct privacy and security risks. Managing HIV data, essential for understanding the epidemic, involves data collection that raises concerns about privacy, consent, and stigmatization [20, 21]. Dunbar and Jones [23] highlight how molecular surveillance data-driven public health measures can exacerbate surveillance of Black communities. Molecular-based surveillance techniques are increasingly used by public health experts to rapidly identify and manage disease outbreaks like HIV by analyzing genetically similar virus strains, enabling more effective intervention and overcoming traditional surveillance challenges [24]. McClelland et al. [24] discuss the growing use of molecular surveillance in public health for managing diseases like HIV and highlight ethical and legal concerns, including privacy and consent issues, especially in Canada’s context of over-policing marginalized groups and criminalizing HIV. Canada, a world leader in criminalizing HIV exposure, transmission, and non-disclosure, also has pervasive criminalization regimes, notably over-policing drug users, sex workers, and individuals with precarious migration status [24]. The criminalization of HIV transmission intensifies discrimination and stigma, deterring individuals from seeking testing and healthcare [25]. The intersection of HIV testing with immigration policies further complicates the landscape by introducing fear and stigma among racialized and ACB migrants and raising human rights issues related to deportation based on HIV status [26, 27].

Importantly, the secondary uses of HIV biomaterials and information remain poorly understood, with the need for transparent biomaterial use being a key ethical issue [28]. Media cases have highlighted ethical issues in consent, privacy, and ownership of biological samples [29, 30]. In recent years, debates in Canada have also centered around the secondary use of health data in public health research, such as the use of identifiable patient data by government health agencies during the COVID-19 pandemic [31]. These examples mirror concerns in HIV testing, where individuals worry about the potential commercialization of their HIV data. Molldrem and Smith [32] examine the bioethical challenges in HIV surveillance and cluster detection, focusing on consent and privacy risks associated with the re-use of clinical HIV data for public health purposes. While using patient data and bio-samples from healthcare for biomedical research, precision medicine, and a learning health system shows promise [33, 34], the ethical implications of secondary HIV data and biomaterial use are not well explored. The scope of patients’ consent is crucial for determining secondary use options [35]. However, there’s a debate among experts in data protection, research ethics, healthcare, patient advocacy, and health insurance over how broadly consent should describe data use purposes [36–38]. Given the challenges in foreseeing all future data uses at the time of initial consent, further research in this area is essential.

This paper explores the ethical ramifications surrounding access to and secondary use of HIV data and bio-samples. By exploring ACB community perspectives, this inquiry is essential to ensuring that these practices not only adhere to rigorous ethical standards but also resonate with the needs and concerns of the impacted communities, fostering a more ethical and just approach in HIV data management.

## Methodology

### Study development and approach

In this Ubuntu-Pamoja study, wherein ‘Ubuntu’ is an African concept that means humanity to everyone while ‘Pamoja’ means together in Swahili, the Community Guiding Circle (CGC) played a crucial role in shaping the research process. The Ubuntu-Pamoja framework was developed specifically for this study to embody community-centered values and collaborative principles. Comprising 10 ACB community members, the CGC actively participated in various stages of the study, including research conception, data analysis, and member checking. The CGC was formed specifically for this study, ensuring representation and inclusion of the community’s perspectives from the outset. They met regularly, both in-person and remotely, to provide valuable community input, ensuring that the study was closely aligned with the perspectives and needs of the community. We also strengthened the capacity of CGC members to conduct HIV research and interventions. The study used a community-based research (CBR) approach. This collaborative approach between researchers and the CGC enriched the study’s design and outcomes.

### Participant recruitment and eligibility

Participants were recruited through flyers at community agencies and peer recruiters and using social media. Eligibility included: (1) identify as a Black African or Caribbean, (2) be 18 years of age or older, and (3) live in Manitoba. All procedures performed this study were approved and were in accordance with the ethical standard of the institutional research committee (University of Manitoba Research Ethics Board; protocol # HE2022-0264). Informed written and verbal consent was obtained from all participants. All data were kept confidential.

### Data collection and analyses

In-person and online interviews lasted one hour, and participants were compensated $40. The semi-structured interviews were conducted by four female ACB research assistants (1 PhD, 3 Master’s RAs with extensive training in qualitative research) and explored participants’ perspectives and experiences with HIV testing, including access to HIV testing. Interview questions were developed for this study (see supplemental file). Reflexive inductive thematic data analysis [39] involved delineating units of meaning from the data, clustering units of meaning to form thematic statements, and extracting themes. Using MAXQDA qualitative software analysis, the research team carefully read through all the transcripts, line-by-line, to identify and annotate initial codes based on their interpretation of the text. Next, the team undertook an iterative process to identify patterns and connections between codes. This involved categorizing codes into preliminary categories based on commonalities and recurring ideas observed during coding. Through reflexive discussions, the team critically examined how these categories represented participants’ perspectives and aligned with the study’s objectives. Key categories emerged, such as ‘mistrust in institutions,’ ‘concerns about data misuse,’ ‘historical exploitation fears,’ and ‘desire for control and transparency.’ These categories captured specific dimensions of participants’ concerns, such as fears of data access by institutions like police and immigration, or apprehensions regarding the commercialization of biosamples. These categories were then mapped to overarching themes that encapsulated broader insights. This mapping process was driven by a reflexive approach, where the research team consistently revisited and refined categories, ensuring they authentically represented participant voices. Finally, the research team refined and named the identified themes. To increase the trustworthiness of the findings, four members of the team did the analysis and then checked for consistency in three research team meeting and four CGC meetings.

### Sample characteristics

Thirty-three ABC community members in Manitoba participated in the study. The participants had a mean age of 34, ranging from 18 to 50 years old. Among them, 20 individuals identified as women, and 13 as men. In terms of sexual orientation, 25 participants identified as heterosexual, while 8 identified as members of the LGBTQIA + communities. Geographically, participants lived in Winnipeg (Manitoba’s largest urban center), and two smaller cities – Brandon and Steinbach. Regarding educational attainment, 27 individuals had some form of university or college education, while six individuals had a high school diploma. Among all participants, 22 were actively employed, 2 were international students, and 3 were unemployed. The study sample included 19 permanent residents, 2 study permit holder, 3 work permit holders, 7 refugees, and 2 Canadian citizens. Immigrants and refugees in the study had lived in Canada for varying lengths of time, with an average of 9.8 years (the range of time spent in Canada varied from six months to 30 years).

### Findings

Study participants expressed mistrust over institutions like police, child welfare, and immigration accessing their health data. Concerns about data misuse, criminalization, and historical exploitation of Black communities in medical research were prevalent. While some were willing to contribute samples for medical science, there was a strong demand for greater transparency and control over their health information. Participants emphasized informed consent, the need for clear communication on sample use, and ethical considerations like compensation for sample use in future research.

### Concerns surrounding access and misuse of HIV-related information by institutions

The majority of participants in the study voiced profound mistrust in how various institutions like police, child welfare, and immigration can access and misuse participants’ health information. Participants expressed dissatisfaction with the limited information provided about the journey of their samples and the potential for immigration services to gain access to their HIV testing results:

“I don’t think they provide enough information. They just tell you the basics of why you are doing the tests, and if the result is positive, how you can be linked to care. Nobody really goes into details regarding how your sample travels and who has access to your results… I don’t know if they would deny me permanent residency because of my HIV status… They may not give you permanent residency or citizenship” (28-year-old Caribbean man).

This quote underscores the fear of participants that their health information might impact their immigration status. This perceived risk reflects a broader concern about the lack of transparency around data access, which can deter people from seeking necessary testing.

Many participants evoked the potential involvement of child and family services, echoing a history of systemic injustices faced by Black communities:

I think that there’s a lack of information around this, especially when it comes to immigration, police, and child and family services. Some people might [say], oh, they’re going to take my kids away from me. So, they weren’t well informed about the process and the consequences of giving samples and the results (34-year-old African man).

This statement highlights the participants’ concerns rooted in historical injustices, suggesting a need for clear communication to address fears about how health data might be shared across institutions. For many, past experiences of discrimination contribute to a strong sense of caution.

Another participant evoked a recent HIV criminalization case in the Canadian media about a refugee living with HIV being deported: “Media too, I am recalling an incident that required the deportation of someone who was HIV positive…and people still talk about it today. I think that’s a reason to fear” (40-year-old African man). Here, the participant connects media stories with personal fears of criminalization and deportation. This connection shows how publicized cases can amplify anxiety among vulnerable communities, making them reluctant to engage in testing due to the potential for stigma and legal repercussions.

The fear of personal data associated with test results being shared across multiple institutions and the potential for criminalization contributed to hesitancy among some community members:

Those who suspect they might be HIV-positive don’t want to go and do the testing to confirm. If one institution has my result, another institution can check these results… Clinics, hospitals, and other establishments that deal with health issues, communicate So, in terms of criminalization, how am I going to prove that I am safe when I have my result shared everywhere? (33-year-old African man).

This quote reflects mistrust and fear of potential criminalization. Many participants felt that sharing health data with multiple institutions could compromise their privacy, resulting in stigmatization and punitive actions.

Respondents were also worried about the consequences of testing on health insurance: “Community members are concerned because it might affect their health insurance premiums because of the number of bills. I think there will be some concern on the part of community members regarding who has access to their sample and their results.” (30-year-old Caribbean woman). This reflects the anxieties associated with health data sharing, where participants feared that their HIV status could negatively impact their insurance rates. This concern highlights a perceived link between medical testing and broader socio-economic consequences, influencing participants’ willingness to seek testing.

### Uncertainty regarding the future use of HIV data and biosamples

The recurring theme among participants in this study was the uncertainty regarding the future use of HIV samples:

I honestly don’t know. I think it would be helpful in one regard in medical a advancements. But on the other hand, you don’t have a lot of control or knowledge of what they will be doing with that sample, so I’m unsure. (36-year-old African woman).

Some participants evoked historical discourse on exploitation of Black communities in medical research and testing:

I think just given the history of testing on Black people and what the outcome of that was, I can see why, you know, Black people are hesitant about it… Also, in the past there have been examples of, you know, that stuff being used against Black people, so I think those concerns are valid. (29-year-old Caribbean woman).

Similarly, one participant commented: “Historically, the black community has been used as Guinea pigs for various types of research, so that is still in our memory… I can definitely see concerns from community members with regards to the future use of their own [blood] samples” (42-year-old African man). Along these lines, some participants talked about their fears of others exploiting their bio-samples:

I do know there have been instances through history where people do take it upon themselves to exploit our samples, our DNA, and things like that, so I have concerns about the future use of my samples. (36-year-old African woman).

This participant’s words reflect a fear of exploitation rooted in both historical precedent and contemporary concerns about misuse. These concerns emphasize the importance of informed consent and transparent policies on how bio-samples will be stored and used to address fears of exploitation.

Some participants were also open to the idea of their health-related information or blood samples being used for further research, reflecting some degree of trust in the medical field. However, concerns about who has access to their test results were voiced by some participants. These concerns primarily revolved around non-medical professionals, such as social workers or public health officers, accessing their information without their consent. Participants expressed a preference for being informed and involved in the decision-making process if access to their information was deemed necessary for non-medical purposes:

I am OK with doctors seeing it in the future, but if, let’s say, a social worker or other public health officers want access to it, for whatever reason then I would like if I was maybe like notified and given the choice of whether or not I would like them to have access to it or not (27-year-old African woman).

This response indicates that while participants may trust medical professionals, they are uncomfortable with the idea of non-medical personnel accessing their information without consent.

Another participant highlighted the ethical boundary concerning the future uses of biomaterials: “If what I am giving is going to help more people… maybe used to find a cure, then that is good and ethical. However, if it goes in a negative direction, causing stigma, then I have a lot of issues with that. We have to show this sample won’t bring fears, won’t stigmatize the community, and won’t be a weapon” (45-year-old Caribbean man).

Similarly, another participant commented:

Is it all destroyed once they give me my results or what happens with my sample? I would need to see what my results were, and if I personally thought that it would be a help to science, then I’d let them keep my results…Once I’ve got my results, I’d like to be able to sign something that says either permission to keep this for future research or permission to destroy this right away” (33-year-old African woman). Participants also stressed the necessity of providing clear information and transparency: “Tell community exactly where the sample is… a path. Even if you have to do a diagram… a picture of where their sample travels. Very straightforward and informative before they agree.” (28-year-old Caribbean woman). Some participants also contemplated the potential use of health-related information or samples collected during an HIV test for future research or studies, for example: “When they do this… after maybe… three or four years… will they still come back to tell me? They can use my sample, but I don’t want to hear about that after like 10 years (24-year-old African man). This statement reflects a concern with long-term storage and future use of samples, illustrating the need for ongoing communication with participants if samples are retained.

Some participants also voiced concerns about the future sale of their information:

I don’t want the information to be sold; I don’t know where people sell to. Everyone is always paranoid about it getting sold to something. I don’t want that. Again, I’m assuming that it [test result] will be treated in an ethical manner” (31-year-old Caribbean woman).

This quote reflects a deep-seated fear among participants about commercialization and loss of control over their data. Lastly, one participant asserted that if researchers were to use their sample in the future, “there should be a consent and probably further compensation” (37-year-old African woman). This highlights the expectation among participants that they should not only be informed but also compensated for any future uses of their samples, reflecting an awareness of the value of their contribution and a desire for reciprocal respect.

## Discussion

This study highlights the ACB communities’ concerns regarding the collection and dissemination of HIV biomaterial and personal health information, reflecting key aspects of structural violence [10, 11]. These concerns, rooted in fears of human rights violations like compromised privacy and bodily autonomy, illustrate the power imbalance in healthcare. Additionally, these concerns align with Farmer’s [10, 11] concept of structural violence in the sense that they highlight racial injustice and systemic issues within healthcare that contribute to ethical challenges. These ethical concerns also emphasize the need to critically examine healthcare practices, especially for Black communities, to promote equitable and just experiences.

The study’s findings align with existing literature on ethical concerns in HIV testing, including confidentiality, privacy, informed consent, discrimination, and mandatory reporting issues [14–17]. They reinforce the literature’s discourse on the structural vulnerabilities of ACB communities to HIV [1, 2, 4–7]. While other literature examined structural elements and factors such as gendered power relations, poverty, lack of information, economic disparities, inadequate socio-economic infrastructure, and limited educational opportunities [40–42], our study contributed to an understanding of the structural barriers to HIV testing among ACB people that are deeply rooted in systemic racism and historical socio-structural inequities, as well as how these structural elements render themselves to have ethical implications.

The study highlights the significance of acknowledging the cultural context and historical experiences of ACB communities in HIV testing. Participants expressed a deep mistrust towards healthcare system. This apprehension, embedded within a structural violence lens [10, 11], illuminates the intricate power dynamics that historically shape healthcare practices and policies. The study calls for clear communication and transparency in HIV testing, focusing on sample handling, test implications, and privacy. In Canada, while there are clear rules regarding data privacy, mandatory reporting, and the limitations on data sharing, such policies are not always well communicated to patients. For instance, there are regulations outlining the circumstances under which HIV data may be shared with public health authorities, but these do not generally extend to non-health entities (such as insurance companies, law enforcement, or immigration authorities). Clear communication about these rules could help mitigate mistrust and alleviate fears around potential data misuse. However, the lack of consistency in data-sharing policies across provinces and territories highlights areas where policy reform could further protect patient rights and enhance trust in the system. Emphasizing informed consent and privacy policies, it addresses fairness issues in healthcare, particularly regarding the impact of HIV testing on immigration, insurance, and legal matters. The study also highlights ethical concerns about discrimination and stigma [41], and advocates for rectifying injustices, creating anti-racist healthcare environments, and ensuring equitable testing [5, 43].

Concerns over the sharing of HIV test results and potential criminalization underscore the importance of confidentiality and trust in testing acceptability [24, 25]. Healthcare providers must follow strict confidentiality protocols to prevent unauthorized HIV status disclosure. Additionally, fears about impacts on immigration and health insurance highlight the need for anti-oppressive, migrant-affirming HIV testing practices and spaces [26]. Viewed through the lens of structural violence [10, 11], this situation further highlights the vulnerability of historically marginalized communities to experiencing criminalization. However, it is also important to note that while not all participants in our study were directly at risk of immigration-related repercussions, many participants still believed they could face negative consequences. This perception of risk is significant, as it may deter individuals from seeking HIV testing due to fears of deportation, stigma, or discrimination.

Participants’ concerns in the study underscore the ethical principles of beneficence and non-maleficence in HIV testing. It’s essential to conduct testing while minimizing potential harms like discrimination or deportation fears. Respecting patient autonomy and informed decision-making is crucial, allowing individuals to control their testing and data use [43]. Balancing confidentiality with public health needs is vital; while maintaining trust and privacy, public health agencies may use de-identified data for surveillance [44]. Clear guidelines and public engagement are necessary to respect privacy rights and enable effective public health interventions [45].

Participants’ concerns about data sharing also underscore the ethical complexities involved in the retrospective analysis of public health data, which is commonly conducted by health departments. While retrospective analyses can enhance understanding of public health trends and improve interventions, these practices must carefully consider participant consent, data anonymization, and transparency. For ACB communities, who already face systemic marginalization and discrimination, retrospective use of data without explicit consent can deepen mistrust and raise fears of potential misuse. Thus, our findings emphasize the importance of establishing clear, community-informed guidelines around retrospective data use to ensure ethical practices that protect participant autonomy and address privacy concerns. Furthermore, policies enabling ACB community representation in decision-making processes regarding public health data could help build trust and foster more equitable public health practices.

Participants in the study expressed deep uncertainty about the future use of their bio-samples, highlighting a tension between contributing to medical advancement and fearing a lack of control over their samples. They navigated ethical considerations between altruism and potential harm, such as marginalization and stigma. This underscores the need for transparent information about biomaterial use to alleviate fears and avoid oppressive practices [28]. Concerns about sample handling and health information reflected a desire for agency and informed consent, emphasizing the importance of clear communication and transparency in healthcare practices to enable ethical decisions.

The structural violence framework [10, 11] also highlighted how societal structures and economic systems contribute to injustices in the realm of potential HIV data commercialization. Participants expressed concerns that economic motivations could overshadow ethical responsibilities, leading to the commodification of their sensitive health information. This profit-driven approach within neoliberal capitalist systems raises ethical questions about consent, control, and benefit sharing for communities contributing to research. By situating these issues within the structural violence framework, our findings underscore the need for policies that protect individuals from exploitation and ensure transparency in the use of HIV data. Participants discussed the commercialization of their HIV data, situating their concerns within the framework of neoliberal capitalism, and expressing worries about profit-driven exploitation and potential ethical issues. In HIV research, innovations like viral load community mapping and HIV strain-specific DNA sequencing [46, 47] offer precision but also risk reinforcing neoliberal logic, societal biases, and deepening inequities, potentially aggravating stigma against racialized and Black communities in economically destitute geographic regions. In response to these issues, Molldrem and Smith [32] advocate for “HIV data justice,” emphasizing the importance of community involvement and data control to address ethical concerns.

HIV testing ethics with ACB communities underlines the need for trust-building in healthcare, emphasizing informed consent and addressing skepticism and exploitation concerns. Our findings stress the need for culturally safe HIV testing for ACB communities in Canada. Community organizations and clinics play a key role in providing safe services, ensuring data privacy, and reducing stigma. Culturally sensitive approaches increase testing acceptability and empower individuals to focus on their health [3, 48]. A comprehensive, decolonizing, and anti-racist strategy is crucial for transforming HIV testing and healthcare into an equitable and just system.

### Study limitations

One key limitation of this study is the use of purposive sampling, which may have biased the results toward the perspectives of community members with existing connections to HIV agencies. This approach, while valuable for recruiting participants within the ACB community, may limit the generalizability of findings, as it may not capture the experiences of ACB individuals who are less connected to these networks or who have different perspectives on HIV testing. Additionally, the relatively small sample size restricts the diversity of voices represented within the study. Although we aimed to capture a range of experiences, the sample may not fully reflect the broader ACB populations in Canada, particularly given the geographic focus on Manitoba. This limits the extent to which findings can be applied to ACB communities in other provinces or regions, where healthcare systems and social contexts might differ. Future research could address these limitations by including larger and more diverse samples across multiple Canadian regions, employing mixed methods to triangulate findings, and exploring different recruitment strategies to reach ACB individuals not currently engaged with HIV services.

## Conclusions

This study sheds light on the ethical challenges surrounding HIV testing within ACB communities in Manitoba, focusing on issues of privacy, informed consent, data ownership, and the potential for data misuse. This research highlights the intersection of historical trauma, structural violence, and systemic racism, which collectively shape the experiences and concerns of these communities regarding HIV testing and data management. The study emphasizes the importance of recognizing these contextual factors to create a more inclusive and respectful healthcare environment.

The findings underscore a need for healthcare policies that prioritize individual rights, particularly around confidentiality, informed consent, and control over personal health data. Participants expressed a clear desire for greater transparency, better communication, and accountability from healthcare providers and institutions, especially in terms of who has access to their data and for what purposes. To build trust, healthcare systems must engage ACB communities in developing and implementing policies that protect privacy and prevent unauthorized data sharing, particularly with non-health entities. Ensuring that ACB individuals have agency over their health information is crucial to overcoming the barriers to testing and reducing stigma.

Future research should build upon these findings by exploring similar issues across other Canadian regions, with a focus on diverse ACB populations and a broader range of healthcare services. These efforts can contribute to a more just and equitable healthcare system that respects the unique histories, needs, and rights of African and Caribbean communities in Canada.

## Electronic supplementary material

Below is the link to the electronic supplementary material.

Supplementary Material 1

## Data Availability

The datasets used and/or analysed during the current study are available from the corresponding author on reasonable request.
