# Supplementary material for: Navigating ethics in HIV data and biomaterial management within Black, African, and Caribbean communities in Canada
Source: BMC Med Ethics. 2025 Jan 16;26:5. doi: 10.1186/s12910-025-01161-0 (PMC11737225; doi:10.1186/s12910-025-01161-0)
Supplement: Supplementary file 1 — Supplementary Material 1 [file 12910_2025_1161_MOESM1_ESM.docx]

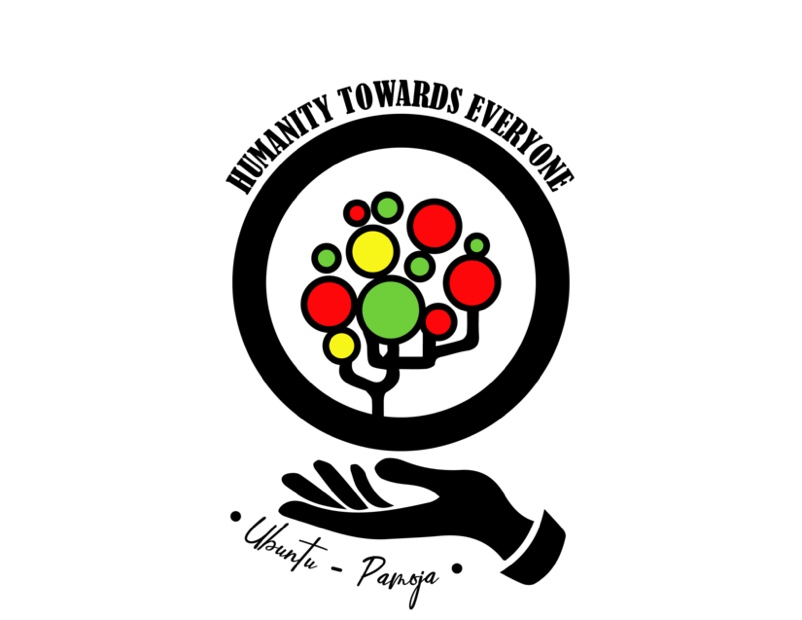

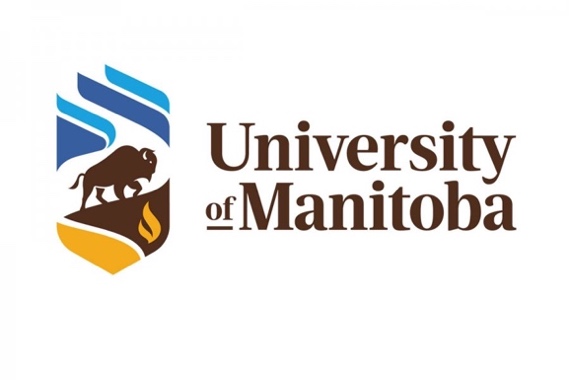


**Interview screening and consent instructions**

Please approach all potential participants with the utmost respect and thoughtfulness. At no point in time should anyone feel coerced into participating in this study or answering a question that they do not want to. Remind the participants of their rights to withdraw at any time. They can withdraw their participation during or after the interview.

State the name and purpose of the study:

- Thank you for taking the time to speak with me. Thank you for participating in our project called “Ubuntu-Pamoja”.
- The Ubuntu-Pamoja Study (HIV/STTBI Testing & The Health of African and Caribbean Migrant Communities in Manitoba) is interested in learning more about your experiences and preferences for HIV and STBBI testing, as well as facilitating Dried Blood Spot testing among members of African and Caribbean migrant communities. Our research tries to explore what approaches to HIV testing (such as dried blood spot) work best for African and Caribbean communities. We also want to create recommendations on developing appropriate programs for these communities. As part of this project, we will ask you questions about HIV/STBBI testing, treatment, prevention, and reducing HIV-related stigma. It is a community-based project, which means members of the African and Caribbean communities are guiding the development and implementation of this project.
- The name *Ubuntu-Pamoja* was chosen in consultation with the members of community guiding circle which consists of Black, African, and Caribbean community members. We have chosen words that resonate with our African and Caribbean cultures: ‘Ubuntu-Pamoja.’ Ubuntu is an African concept that refers to acting in ways that benefit the community (Humanity to others/everyone). Its origin is south Africa. However, the idea commonly runs through African and Caribbean cultures. Pamoja means “Together” in Swahili. We were also inspired by the saying of one of the prominent African leaders Archbishop Desmond Tutu, “we are connected, and what each of us do affects the whole of humanity." That is why we ended up with this name.
- You have been invited to participate in this study because we are interested in learning more about you, and your experiences, perspectives, opinions, and views relating to the HIV testing process in Manitoba.
- I’d like to remind you that your responses will be confidential and used as data for this research study only. Also, please keep in mind that you do not have to answer any questions that make you feel uncomfortable. Just let me know if you feel uneasy, and we can move on to another question.
- Do you have any questions? [wait for verbal affirmation]
- The interview will last approximately 1.5 hours. Once you have participated in the interview, we will give you $40 compensation for your time. The discussion is confidential. We will not ask for your name or any information that will identify you, thus, no identifying information will be connected to the transcript of the interview discussion. The Interview will be audio-taped and then transcribed. Only the research assistant (give your name) and the research advisor (his name is Rusty [Dr. Rusty Souleymanov]) will have access to these tapes and associated transcripts. Your involvement is voluntary, and you can withdraw at any time.

- Would you be interested in participating in this interview?

If they are interested, please then explain that we have a few screening questions to make sure they are eligible to participate.

The screening questions are:

- Are you 18 years of age or older?
- Do you identify as African, Caribbean, or Black?
- Do you currently live in Manitoba?
- Would you feel comfortable answering personal questions about your health and wellbeing, access to health care, health status, experiences of oppression, and experiences with low-threshold services in Manitoba?

If they are eligible, please have them review verbally and in writing the consent form and mark the form (an actual signature is not required of participants, and an “X” mark is enough).

**Warm up Question:**

- How are you doing? How is your day going?

**Demographic Questions for Community Members**

- How old are you?
- How long have you lived in Manitoba?
- What gender do you identify as?
- How do you identify in terms of your ethnicity?
- What is the highest level of education you completed?
- Current employment or community role: Do you currently have paid employment? What is your occupation?

**Questions on HIV/STBBI Testing (including DBS):**

1. **Have you ever been tested for HIV or STBBI? *(Provide interviewee with a definition of STBBI or HIV if needed)***
   1. What do you know about HIV or STBBI testing?
   2. Do you know how or where to access HIV or STBBI testing in Manitoba?
   3. What do you think are the gaps with regards to HIV or STBBI testing in Manitoba?
   4. What works well for you in terms of testing in Manitoba?
2. **What do you know about Dried Blood Spot (DBS) testing? *(Provide interviewee with a definition of DBS if needed)***
   1. Do you think you need more information about DBS testing? (For example: evidence on the safety of the DBS test; support for people taking the DBS test)
   2. Do you know how to access the DBS test?
   3. Do you know how to self-sample for the DBS test?
      1. What kind of support would you need to perform DBS self-sampling?
3. ***Provide information on how DBS testing allows individual to do self-sampling at home****.* **What does it mean to you to have access to testing in the place of your choice (for example home)?**
   1. What do you think about DBS self-collection at home?
   2. Do you think there could be any issues with self-sampling at home?
   3. Do you think there could be any issues with delivery of tests to your home/residence?
   4. Would it be OK to pick up the DBS sampling kit at a clinic/agency and then bring it home to do your test at home?
4. **Where (location-wise) would you find it safe to be tested for HIV or STBBI in Manitoba?**
   1. What about specific locations for testing? (*Ask them to name clinics, agencies, and organizations that they think are safe for testing).*
5. ***Explain how DBS allows to be tested for multiple health issues (HIV, Hepatitis C, syphilis).*** **If you were able to use the same DBS sample to test for multiple health issues (HIV, Hepatitis C, syphilis, COVID-19), would you like to be tested using this combination approach?**
   1. What does it change in terms of your willingness to take this test?
   2. Is this approach more or less appealing?
6. **Where would you prefer to do the DBS test?** *Give examples: at home, or in a clinic/hospital, community center/agency.*
7. **What do you know about self-testing? *(Provide interviewee with a definition of self-testing if needed)***
   1. Do you think you need more information about self-testing testing? (For example: support for people taking the self- test, counselling)
   2. What kind of support would you need to perform self-testing?
8. **Do you prefer supervised or unsupervised testing?**
   1. Would you be willing to allow a trained local person in your community (we call them *peer navigators*) to collect your blood for a DBS sample? **(*Explain what peer navigator means if necessary).***
   2. Which kind of person would you like to do this type of work in your community?
      1. What are the preferred qualities that persons/peer navigators would possess?
9. **Would you be willing to go for confirmatory testing if necessary? (*Explain what confirmatory testing means).***
   1. What would make it easier to go for confirmatory testing?
   2. ***Explain what phlebotomy means and how invasive it is.*** Would you be receptive to phlebotomy if you are reactive through DBS testing?
10. **Would you want to know the result of your test?**
    1. Why? Why not?
11. **How do you want to be notified regarding your results? Would you want someone to give you a call, would you prefer going to the clinic, or receive results by mail or virtually?**
12. ***.*** **Would you be interested in linkage to care if your screening test is reactive for HIV, or hepatitis C or syphilis?**
    1. What would make it easier for you to reach out to service providers if you are diagnosed?
    2. If you need to be linked to care, do you know where to go?
13. ***Explain what post-test counseling means.*** **Would you prefer post-test counseling?**

***Thank you for your answers.***

***I have a more questions for you. Are we OK to proceed?***

**Questions on Safety & Confidentiality:**

1. What information (e.g., name) would you be comfortable sharing with the service provider if you go for testing at a health clinic/agency?
   1. Are you concerned about confidentiality? Why?
2. Are you concerned about the use of your samples and health information that is collected during testing?
   1. Are you concerned where it travels, what happens with it, who has information to your health-related answers or blood sample? Why?
   2. Would you be OK if public health researchers use the health-related answers or blood sample that is collected from you when you go for testing in their research projects in the future, or would you want that sample to be destroyed after you get your test result?
   3. Do you have any concerns about who can have access to your results?
   4. Do you think community members may be concerned who has access to their results? (for example: Public Health, Social Workers, Immigration Canada, Police, Child and Family Services, Health Insurance, etc)?
      1. Do you think the Black, African, Caribbean community members have enough information about where their blood and health information travels after they do HIV or STBBI test in Manitoba?
3. What are your suggestions for supporting community members to feel confident about our research and the protocols?

***Thank you for your answers.***
